# Thermal Infrared Anomalies of Several Strong Earthquakes

**DOI:** 10.1155/2013/208407

**Published:** 2013-10-10

**Authors:** Congxin Wei, Yuansheng Zhang, Xiao Guo, Shaoxing Hui, Manzhong Qin, Ying Zhang

**Affiliations:** ^1^Lanzhou Institute of Seismology, China Earthquake Administration, Lanzhou 730000, China; ^2^Lanzhou Base of Institute of Earthquake Prediction, China Earthquake Administration, Lanzhou 730000, China; ^3^Earthquake Administration of Liaoning Province, Shenyang 110034, China

## Abstract

In the history of earthquake thermal infrared research, it is undeniable that before and after strong earthquakes there are significant thermal infrared anomalies which have been interpreted as preseismic precursor in earthquake prediction and forecasting. In this paper, we studied the characteristics of thermal radiation observed before and after the 8 great earthquakes with magnitude up to *Ms*7.0 by using the satellite infrared remote sensing information. We used new types of data and method to extract the useful anomaly information. Based on the analyses of 8 earthquakes, we got the results as follows. (1) There are significant thermal radiation anomalies before and after earthquakes for all cases. The overall performance of anomalies includes two main stages: expanding first and narrowing later. We easily extracted and identified such seismic anomalies by method of “time-frequency relative power spectrum.” (2) There exist evident and different characteristic periods and magnitudes of thermal abnormal radiation for each case. (3) Thermal radiation anomalies are closely related to the geological structure. (4) Thermal radiation has obvious characteristics in abnormal duration, range, and morphology. In summary, we should be sure that earthquake thermal infrared anomalies as useful earthquake precursor can be used in earthquake prediction and forecasting.

## 1. Introduction

According to limited infrared remote studies, there is abnormal radiation anomalies before great earthquakes, which is a natural phenomenon that happened during the earthquake process, and it is the basis of our study. With the development of satellite remote sensing technology, the studies on relation between satellite infrared remote sensing and earthquakes can date back to 1980s [1–9]. Recently, many countries in the world have research on earthquake thermal radiation, including China, which has made great progress in its extraction methods [10–20].

As we know, extracting seismic radiation anomaly is very important for the research on seismic thermal radiation, because the anomaly signal is weak and often interfered by the complicated background information. In this paper, we effectively removed the non-seismic anomaly factors and highlighted thermal radiation of seismic anomalies by using the method of “time-frequency relative power spectrum,” which is easy and convenient to operate and reduced by experiences identifiable differences [21–24].

In this paper, we applied the satellite infrared remote sensing data (quite black-body radiation, for short TBB) from Chinese Geostationary Meteorological satellite *FY-2C/2E* to study the changes of thermal radiation observed before and after the eight great earthquakes. Firstly, we obtained the time-frequency space data in dominant frequency and amplitude after the treatment of wavelet transform and relative power spectrum. Then, we gained the seismic radiation anomalies by scanning the time-frequency data with the method of time-frequency map in whole space-time and full band.

## 2. Data Processing Methods

There are several steps for data processing. The first step is to select the types of satellite infrared remote sensing data. For earthquake research, Geostationary Meteorological Satellite data is more suitable than Polar Orbit Satellite in contrast of consistency. As we know, ground feature thermal radiation intensity received satellite infrared remote sensing sensor is principally influenced by temperature and emissivity of object, in addition to atmospheric effects on transmission. Geostationary Meteorological Satellite has advantage in time consistency (the service is midnight's observation data every day and the time-gap is 30 minutes) and place comparability (for the same location, satellite's azimuth, and path of radiation-propagation is almost the same). Hence, we select Geostationary Meteorological Satellite infrared remote sensing data as the data source. The satellite data used in this paper is taken from the China Geostationary Meteorological Satellite (*FY-2C/FY-2E*) infrared remote sensing brightness temperature data, which is received from station of Geostationary Meteorological Satellite at Lanzhou National Geophysical Research Observatory Station of Lanzhou Institute of Seismology, China Earthquake Administration and Satellite Meteorological Center of China Meteorological Administration. The *FY-2C* satellite was launched in 2004 and positioned at 105°E above the equator. In 2008, The *FY-2C* satellite was replaced with* FY-2E* because of meeting design life. The satellite can observe about one-third of the area of the Earth. The time-gap is 30 minutes or one hour, which can cover the entire China and the peripheral areas. The substellar point of infrared resolution is 5 km. The time range of the effective data used in this paper is from April 1, 2006, to March 31, 2011. We took five observation data at midnight every day because there is little solar radiation in the night. The partial cloud influence was removed by the simple treatment with making up the window law, and then we calculated the mean value of these data to obtain the labor-day wage [21]. 

### 2.1. Brightness Temperature Data Processing

The Planck Radiation Law describes the spectral distribution of blackbody radiation, and the formula is

(1) $$\begin{array}{c} B\left(v,T\right)=2hc^{2}v^{3}{\left(e^{chv/kT}-1\right)}^{-1}, \end{array}$$

where *B*(*v*, *T*) is the spectral radiation remittance (w/m^2^ · *sr*⁡·cm^−1^) for wave-number *v* (cm^−1^) and temperature *T* (K), *h* is Planck Constant, *c* is the velocity of light, and *k* is Boltzmann Constant.

Stefan-Boltzmann's Radicalization Law describes the relationship between integral radiation of blackbody and temperature, and the formula is

(2) $$\begin{array}{c} E_{T}=\sigma T^{4}, \end{array}$$

where *σ* is Stefan-Boltzmann's Constant, *σ* = 5.6696 × 10^−8^ W · m^−2^ · K^−4^.

Consequently, we indirectly gained the temperature information of object by measure of radiation energy on the surface or by use of thermal remote sensing image data. However, as the influence of emissivity, we difficultly gained the true temperature of real surface.

The satellite infrared remote sensing wave band observation data are firstly radiometric calibrated and geometric corrected to obtain wave band radiation remittance. Then we calculate the corresponding relation between radiation remittance and temperature for each wave band for temperature. Finally, we can obtain the corresponding temperature of blackbody radiation, which is the brightness temperature BT (K).

### 2.2. Wavelet Transform (WT) and Relative Power Spectrum (RPS) Estimating

The second and most important step for data processing is selecting methods and theories of calculating. In this paper, we used wavelet transform (WT) and relative power spectrum (RPS) estimating for treatment of Geostationary Meteorological Satellite brightness temperature data. We separated the basic earth temperature field (direct circulating part), annual variation temperature field, daily variation temperature field, temperature changes caused by the rain clouds, cold-heat air currents, and other factors (including earthquakes) with the method of wavelet transform (WT). We also obtained the time-frequency space data with the dominant frequency and amplitude after the wavelet transform and the treatment of the relative power spectrum (RPS).

#### 2.2.1. Wavelet Transform (WT)

Wavelet transform known as the “mathematical microscope,” widely used in the field of numerical signal processing and developed very fast because of high resolution in time and frequency domains. The most important advantage of wavelet transform is the nonfixed time window. We have adopted a long time window when accurate low-frequency information, and vice versa. 

**Figure 1:**
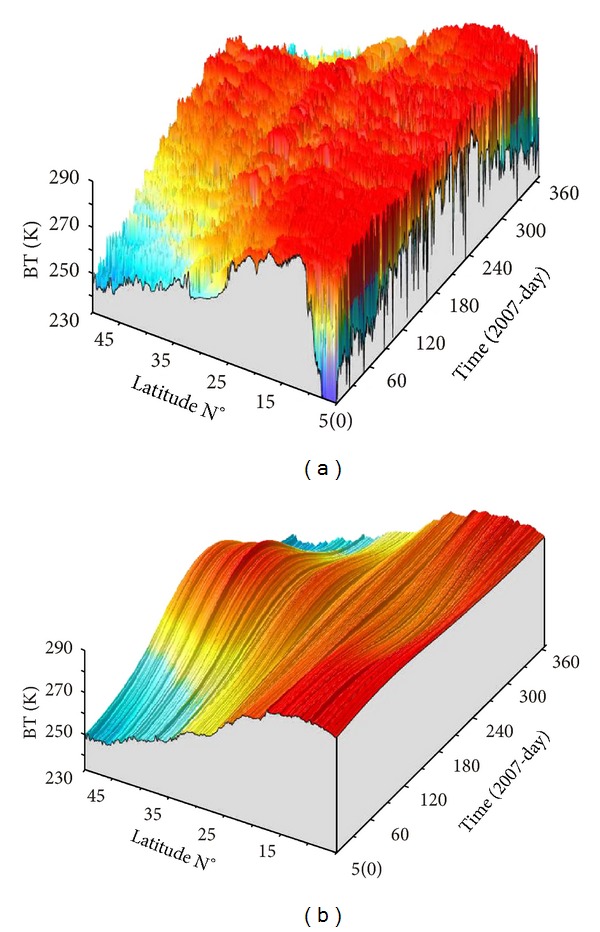
Wavelet transform (WT) result. (a) Raw data; (b) wavelet transform data of seven order criterion function cancellation.

As we know, continuous brightness temperature data includes basic earth temperature field (direct circulating part), annual variation temperature field, daily variation temperature field, temperature changes caused by the rain clouds, cold-heat air currents, and other factors (including earthquakes). So, we used the following steps for calculating thermal radiation brightness temperature data from satellite thermal infrared remote sensing data. Firstly, we choose observation data of midnight in local time to avoid the influence from solar radiation. Secondly, we used the method of wavelet transform for calculating the labor-day wage data after treatment with making up the window law. The basic earth temperature field and the annual variation temperature field can be removed through Wavelet Transform by eliminating the wavelet seven order part. At the same time, the short scale of temperature change caused by rain clouds and cold-heat air currents can be efficiently eliminated after Wavelet Transform by rounding off the details of wavelet second-order part. Lastly, we gained a waveform of brightness temperature data (second order criterion function and seven order criterion function cancellation) with positive and negative phases in time domain for each pixel [21]. The results of Wavelet Transform showed that we can gain the background of brightness temperature along the 105°E during the year 2007 (see Figure 1 on the right).

#### 2.2.2. Relative Power Spectrum (RPS) Estimating

Signal spectrum analysis is an important method for studying the characteristics of signal. For deterministic signals, Fourier transform can be used to examine the nature of its spectrum. Moreover, power spectrum reflects the distribution of power energy of the random signal frequency components. It can reveal the useful information of hidden signal periodic and the close proximity of the peaks.

**Figure 2:**
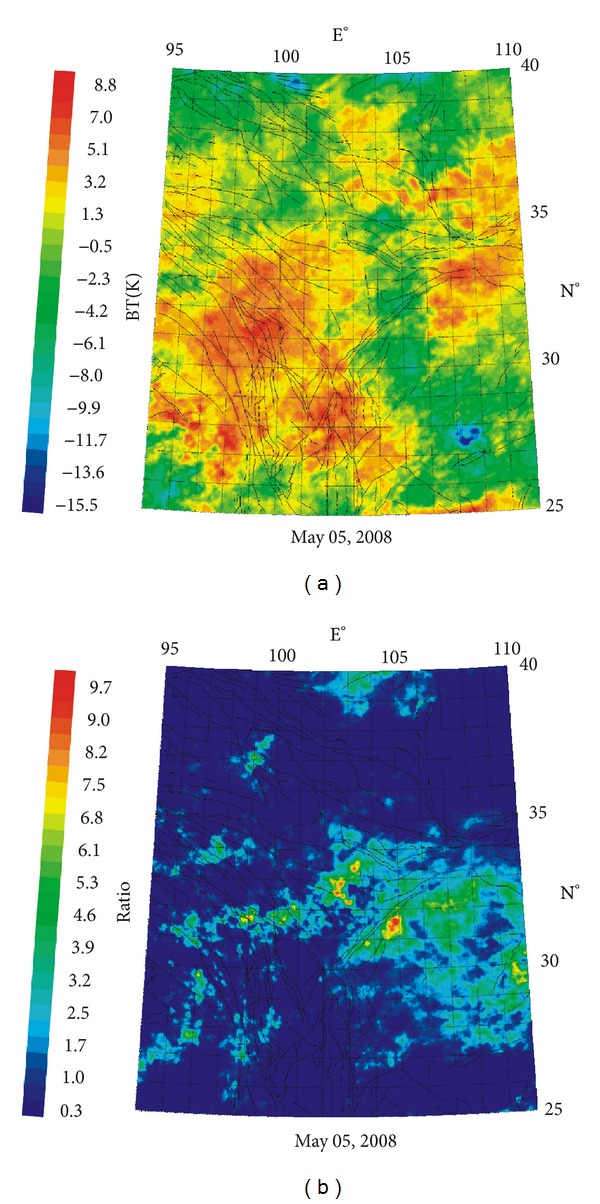
Relative power spectrum (RPS) result ((a) wavelet transform data; (b) relative power spectrum data).

Using the relative power spectral (RPS) method, we can obtain the dominant frequency and the peak-to-peak amplitude value. Firstly, we calculate the power spectrum with fast fourier transformation using a time window length of *n* = 64 (day) and sliding window length of *m* = 1 [21]. We can obtain one-group of power spectra through sliding time-histories data one time for each pixel. Then, we used the relative processing method for every pixel [22]. Lastly, we can gain the time frequency and space data. The picture on the right showed that, the time-frequency and space information before Wenchuan *Ms*8.0 earthquake are obvious and accurate (see Figure 2).

### 2.3. Information Extraction Method of Earthquake Thermal Infrared Anomalies

The information extraction method of earthquake thermal infrared anomalies in this paper is time-frequency map. We effectively removed the nonseismic factors and highlighted earthquake thermal infrared anomalies by scanning the time-frequency space data with the method of time-frequency map in whole space time and full band. It is also the purpose of extracting the information of earthquake thermal infrared anomalies.

**Figure 3:**
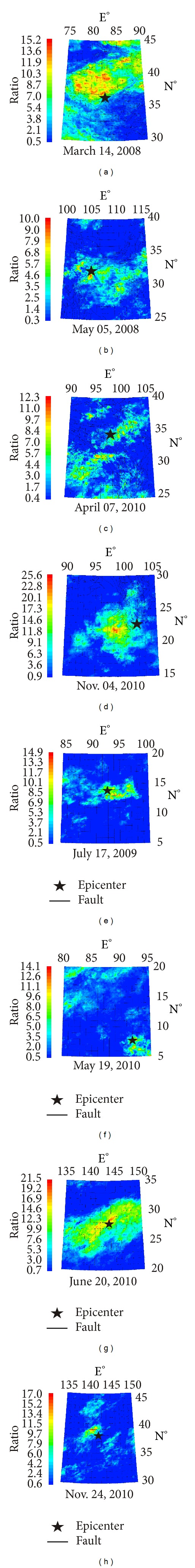
Information extraction result of earthquake thermal infrared anomalies ((a) Yutian *Ms*7.3 earthquake; (b) Wenchuan *Ms*8.0 earthquake; (c) Yushu *Ms*7.1 earthquake; (d) Myanmar *Ms*7.2 earthquake; (e) Andaman Islands *Ms*7.5 earthquake; (f) Nicobar Islands *Ms*7.6 earthquake; (g) Bonin Islands *Ms*7.4 earthquake; (h) Northeast Honshu *Ms*9.0 earthquake).

We effectively gained the characteristics of earthquake infrared anomalies through the information extraction methods. In Figure 3, there is extraction information of earthquake thermal infrared anomalies before 8 earthquakes. Firstly, earthquake occurred the around and the edge of its maximum anomalies. Secondly, anomalies occurred during several days to six months before earthquakes. Lastly, the maximum value of the anomalies is more than 10 times.

## 3. Characteristics Analysis of Strong Earthquakes Thermal Infrared Anomalies

In this paper, we analyzed the characteristics of thermal infrared anomalies of 8 strong earthquakes (4 continent and 4 sea earthquakes; see Table 1) with magnitude up* Ms*7.0 through the wavelet transform-relative power spectrum estimating and time-frequency map scanning. There are several anomalies characteristics of earthquake thermal infrared, such as characteristic period and amplitude, anomalies distribution, and anomalies appeared time (see Table 2). 

### 3.1. Characteristics of Thermal Infrared Anomalies of Continent Earthquake

In this part, we showed the characteristics of thermal infrared anomalies of only two continent earthquakes because the articles of Wenchuan and Myanmar earthquakes have published [21, 24].

#### 3.1.1. Characteristics of Yutian *Ms*7.3 Earthquake Thermal Infrared Anomalies

**Figure 4:**
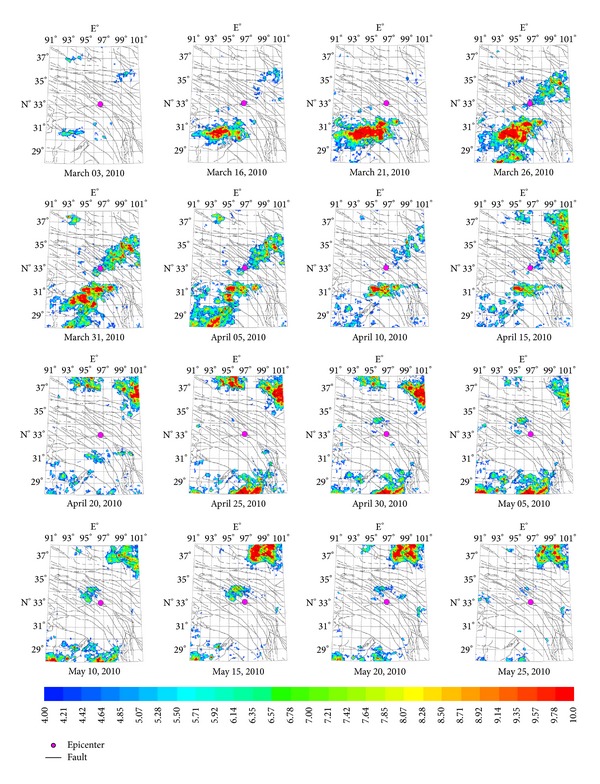
Space-time evolution of Yushu *Ms*7.1 earthquake.

The hypocenter of Yutian *Ms*7.3 earthquake is located on the secondary fault at the joint region between Southern Altyn Tagh fault and Kangxiwa fault of NW striking. Earthquake thermal anomalies were mainly distributed north to the epicenter within the Tarim basin. The characteristic period of the anomalies is about 64 days, and the amplitude of power spectrum is the maximum value (Figure 8(a)) over the five years, which is approximately 16 times than mean value.  Figure 4 demonstrates the characteristic of thermal infrared brightness temperature distribution before and after the earthquake within about two months. The obvious anomalies appeared on March 7 and the anomalies continued until the middle of April and then began to shrink to the microepicenter region and disappeared at the end of June.

#### 3.1.2. Characteristics of Yushu *Ms*7.1 Earthquake Thermal Infrared Anomalies

**Figure 5:**
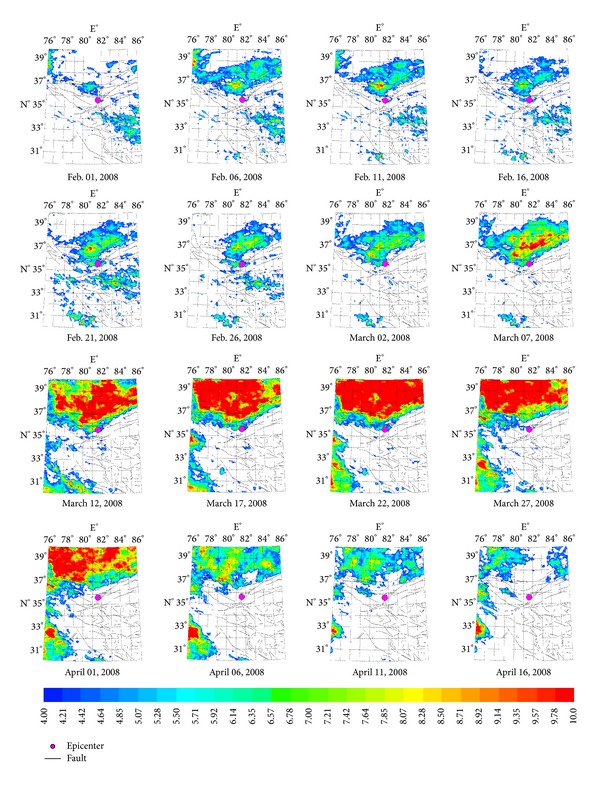
Space-time evolution of Yutian *Ms*7.3 earthquake.

On April 14, 2010, a strong earthquake with magnitude *Ms*7.1 occurred at Yushu County, Qinghai in China. The most obvious anomalies were at the south and northern fracture zone. The characteristic period of the anomalies is about 13 days, and the amplitude of power spectrum is the maximum value (Figure 8(c)) over the past five years, which is approximately 14 times than mean value. The earthquake occurred on the 11th day after the maximum value (observed on May 1).  Figure 5 demonstrates the characteristic of thermal infrared brightness temperature distribution before and after the earthquake within about one month. The obvious anomalies appeared on April 25. The anomalies continued until the end of March and then began to shrink to the microepicenter region and disappeared at the end of May.

### 3.2. Characteristics of Thermal Infrared Anomalies of Sea Earthquake

In this part, we showed the Characteristics of thermal infrared anomalies of only two sea earthquakes because the articles of Andaman Islands and Northeast Honshu earthquakes have been published [23, 24].

#### 3.2.1. Characteristics of Nicobar Islands *Ms*7.6 Earthquake Thermal Infrared Anomalies

**Figure 6:**
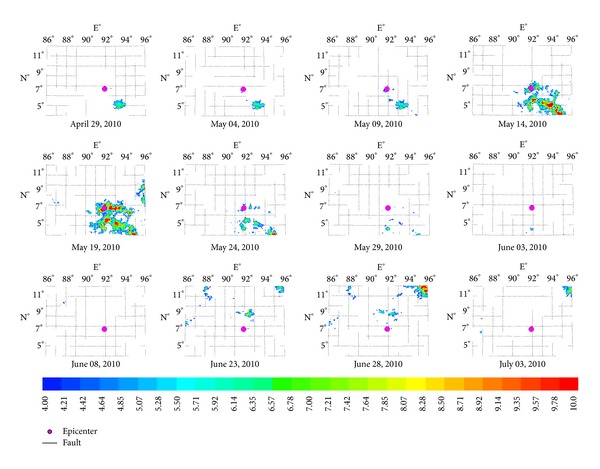
Space-time evolution of Nicobar Islands *Ms*7.6 earthquake.

On June 13, 2010, a strong earthquake with magnitude *Ms*7.6 occurred at Nicobar Islands, India. The characteristic period of the anomalies is about 16 days, and the amplitude of power spectrum is the maximum value (Figure 8(f)) over the past five years, which is approximately 11 times than mean value. The earthquake occurred on the 40th day after the maximum value (observed on May 19).  Figure 6 demonstrates the characteristic of thermal infrared brightness temperature distribution before and after the earthquake within about one month. The obvious anomalies appeared on May 4. The anomalies continued until the middle of June and then began to shrink to the microepicenter region and disappeared at the end of June.

#### 3.2.2. Characteristics of Bonin Islands *Ms*7.4 Earthquake Thermal Infrared Anomalies

**Figure 7:**
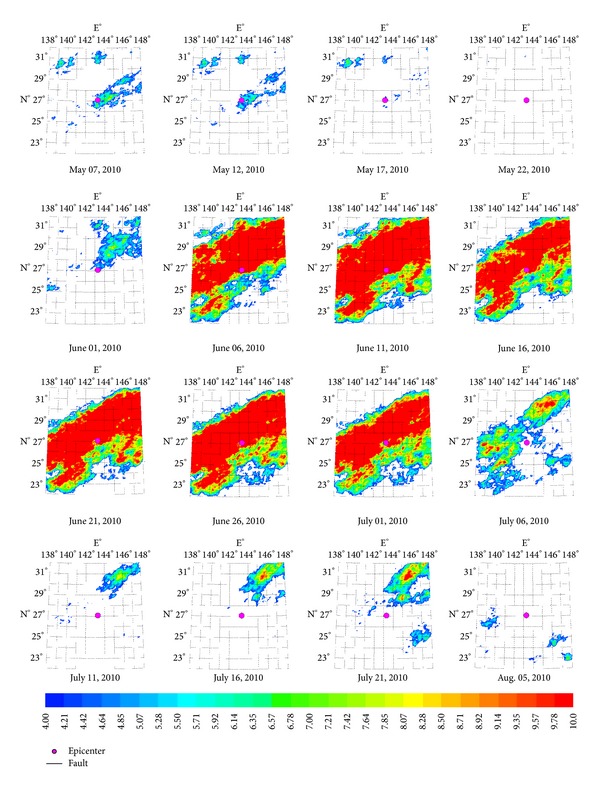
Space-time evolution of Bonin Islands *Ms*7.4 earthquake.

**Figure 8:**
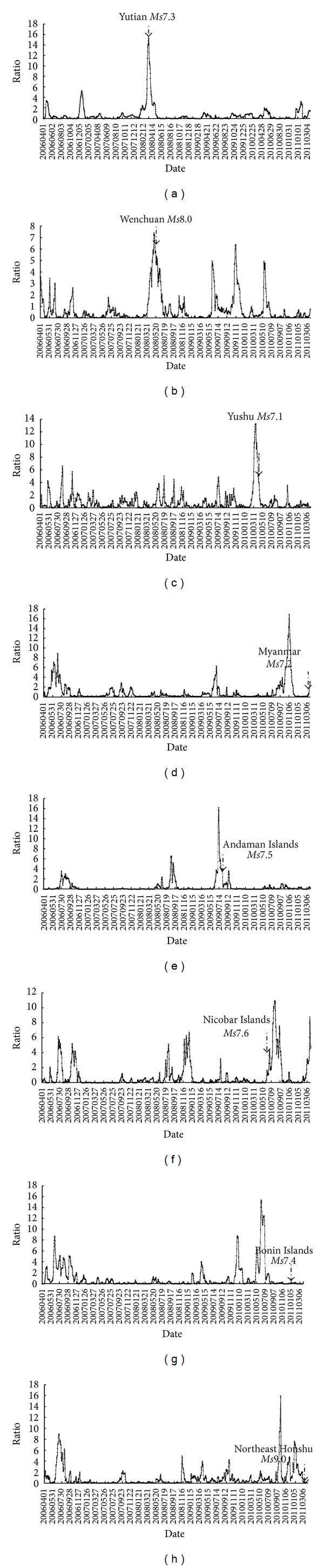
Time series curve of earthquakes. ((a) Yutian *Ms*7.3 earthquake, characteristic period of the anomalies is about 64 days; (b) Wenchuan *Ms*8.0 earthquake, characteristic period of the anomalies is about 13 days; (c) Yushu *Ms*7.1 earthquake, characteristic period of the anomalies is about 13 days; (d) Myanmar *Ms*7.2 earthquake, characteristic period of the anomalies is about 13 days; (e) Andaman Islands *Ms*7.5 earthquake, characteristic period of the anomalies is about 16 days; (f) Nicobar Islands *Ms*7.6 earthquake, characteristic period of the anomalies is about 32 days; (g) Bonin Islands *Ms*7.4 earthquake, characteristic period of the anomalies is about 64 days; (h) Northeast Honshu *Ms*9.0 earthquake, characteristic period of the anomalies is about 13 days).

On December 22, 2010, a strong earthquake with magnitude *Ms*7.4 occurred at Bonin Islands, Japan. The characteristic period of the anomalies is about 13 days, and the amplitude of power spectrum is the maximum value (Figure 8(g)) over the past five years, which is approximately 16 times than mean value. The earthquake occurred on the 180 days after the maximum value (observed on May 7).  Figure 7 demonstrates the characteristic of thermal infrared brightness temperature distribution before and after the earthquake within about two months. The obvious anomalies appeared on June 6. The anomalies continued until the end of June, and then began to shrink to the micro-epicenter region and disappeared at the end of July.

### 3.3. The Results of Characteristics Analysis

The characteristic of earthquake thermal infrared anomalies is obvious and useful in characteristic period and amplitude, anomalies distribution, and anomalies appeared time. Firstly, the characteristic amplitude and periods of such 8 earthquakes are quite different. Secondly, the region where earthquakes occurred is located at the edge or active fault of abnormal area, and the time when earthquakes occurred is within 6 months after the maximum amplitude of power spectrum. Thirdly, it is difficult to analyze the relationship between the earthquake magnitude and the anomalies from those earthquake cases. It is possible that earthquake magnitude is related to the size of the abnormal regional, the abnormal amplitude, the regional geological environment, and the regional atmospheric environment, which should be a very complicated issue.

## 4. Conclusions and Discussions

### 4.1. Conclusions

There are obvious thermal anomalous characteristics (characteristic period and amplitude) before and after 8 great earthquakes. The region in which earthquakes occurred is located at the edge or active fault of abnormal area, and the time is within 6 months before and after the maximum amplitude of power spectrum. The distribution of anomalies and time scales of earthquake occurrence found in this study is consistent with previous studies (several days to 6 months) (see Table 2). 

Based on the analyses of earthquake cases, we got the results as follows.

1. There are significant thermal radiation anomalies before and after earthquakes for all cases. It is easy to extract and identify such seismic thermal radiation anomalies by using of “the relative power of time-frequency spectrum.” The overall performance includes two main stages: expanding first and narrowing later. It may be the result of warming and cooling. The warming is due to a rapid increasing and accumulation of regional stress, and the atmosphere at bottom is rising, which is caused by the greenhouse gas emissions during fault slipping. The cooling of ground (sea) air temperature is caused by atmospheric conditions.
2. There exist evidently characteristic periods and magnitudes of thermal abnormal radiation before and after earthquakes. In terms of the continent earthquakes, there is a long cycle in drought areas, but shorter in wet areas. The characteristic period of mostly oceanic earthquake is 30 days or less. The characteristic magnitude is more than 8 times of normal.
3. Thermal radiation anomalies are closely related to the geological structure. The abnormal distribution and geological structure (fault strike) are basically consistent. Seismic radiation anomaly varies differences in different region and latitude, such as between sea and land, wet areas and dry regional.
4. Thermal radiation has obvious characteristics in abnormal duration, range, and morphology. The duration is between one and six months and varies in different regions. There are significant differences in oceanic and continental earthquakes. Earthquakes occurred within or at the edge of the abnormal transitive region where anomalies appeared and disappeared. The thermal infrared anomalies appeared repeatedly before the majority of earthquakes.

### 4.2. Discussions

In this paper, we should be sure that thermal infrared anomalies as useful earthquake precursor can be used in earthquake prediction and forecasting. On the other hand, in order to improve the ability of earthquake prediction and forecasting by using satellite infrared remote sensing information, there are still many problems which must be solved, because of the complexity formation mechanism, such as unusual earthquake time trends and summaries on a large scale of massive earthquake cases.

Generally speaking, the size of thermal anomaly areas is quite different, but it has nothing to do with the magnitude of the earthquakes. It may be due to the geological, atmospheric, and environmental conditions. According to our research, the earthquake thermal anomalies areas are usually relatively large and are at least tens of thousands of square kilometers. Therefore, we can predict earthquake by using the methods not only mentioned in this paper, but also with the further determination of active tectonics. 

## Figures and Tables

**Table 1:** Earthquake catalog.

No	Date GMT+8	Latitude N°	Longitude E°	Magnitude *Ms *	Depth Km	Location	Source*
1	2008-03-21	35.6	81.6	7.3	33	Yutian, China	CSIN
2	2008-05-12	31.0	103.4	8.0	33	Wenchuan, China	CSIN
3	2010-04-14	33.1	96.7	7.1	14	Yushu, China	CSIN
4	2011-03-24	20.8	99.8	7.2	20	Myanmar	CSIN
5	2009-08-11	14.1	92.9	7.5	33	Andaman Islands, India	CSIN
6	2010-06-13	7.7	91.9	7.6	35	Nicobar Islands, India	CSIN
7	2010-12-22	27.1	143.3	7.4	14	Bonin Islands, Japan	CSIN
8	2011-03-09	38.3	142.6	9.0	20	Northeast Honshu, Japan	CSIN

*Seismic parameters derived from the China Seismic Information Network (CSIN).

**Table 2:** The results of characteristics analysis.

No	Location	Characteristic period/day	Maximum abnormal appearance time	Duration/day	Maximum
1	Yutian, *Ms*7.3	64	1 day before earthquake	60	15
2	Wenchuan, *Ms*8.0	13	4 days before earthquake	80	8
3	Yushu, *Ms*7.1	13	20 days before earthquake	60	14
4	Myanmar, *Ms*7.2	13	170 days before earthquake	90	17
5	Andaman Islands, *Ms*7.5	16	30 days before earthquake	70	17
6	Nicobar Islands, *Ms*7.6	32	40 days after earthquake	90	11
7	Bonin Islands, *Ms*7.4	16	180 days before earthquake	60	16
8	Northeast Honshu, *Ms*9.0	13	130 days before earthquake	90	16
